# Effect of Severe External Airborne Agents’ Exposure on Dementia

**DOI:** 10.3390/jcm9124069

**Published:** 2020-12-17

**Authors:** Seunghyun Lee, Joon Yul Choi, Jin-Ha Yoon, Wanhyung Lee

**Affiliations:** 1 The First Department of Internal Medicine, School of Medicine, University of Occupational and Environmental Health, Kitakyushu, Fukuoka 807-8555, Japan; jihyun6547@naver.com; 2 Epilepsy Center, Neurological Institute, Cleveland Clinic, Cleveland, OH 44195, USA; jychoi717@gmail.com; 3 Department of Preventive Medicine, Yonsei University College of Medicine, Seoul 03722, Korea; 4 The Institute for Occupational Health, Yonsei University College of Medicine, Seoul 03722, Korea; 5 Department of Occupational and Environmental Medicine, Gil Medical Center, Gachon University College of Medicine, Incheon 21565, Korea

**Keywords:** external airborne agents, cognitive function, dementia, National Health Insurance Service in Korea

## Abstract

The impact of occupational and environmental exposure to external airborne agents on cognitive function, especially in incidence of dementia, is understudied. The present study was conducted to elucidate the association between severe external airborne agents’ exposure and incidence of dementia among an elderly population and to explore the effects of exposure to severe external airborne agents on preclinical dementia using the screening test of dementia. From the National Health Insurance Service-Health Screening Cohort (NHIS-HealS, 2002–2015), 514,580 participants were used for data analysis. We estimated the standardized incidence ratio (SIR) according to the exposure to external airborne agents. Of the total participants (*n* = 514,580), 1340 (0.3%) experienced severe external airborne agents exposure, and 26,050 (5.1%) had been diagnosed with dementia. The SIRs (95%CI) of dementia in Alzheimer’s disease, vascular dementia, dementia in other diseases, and unspecific dementia were 1.24 (1.01–1.49), 0.88 (0.37–1.32), 1.16 (0.01–2.77), and 0.69 (0.36–1.02), respectively. The risk of testing positive in the dementia screening significantly increased with exposure to severe external airborne agents after adjusting for all confounding variables. This study found that exposure to severe external airborne agents is a potential risk factor for dementia, especially in Alzheimer’s disease. It is essential to create international awareness regarding the effect of airborne agents’ exposure on dementia.

## 1. Introduction

Exposure to external airborne agents has become a serious issue, as it is a threatening health hazard. For many decades, previous research has explored the relationship between exposure to external airborne agents and its effects on human health [1,2,3]. Inhalation is the most primary route of exposure to external airborne agents, resulting in dust-related respiratory diseases and disabilities, such as chronic respiratory diseases, asthma, chronic obstructive pulmonary disease, chronic bronchitis, lung cancer, and pneumoconiosis [4,5,6].

Pneumoconiosis, an external airborne agents’ exposure-related lung disease, is a class of interstitial lung disease caused by external airborne agents found in the occupational environment that are breathed in and then deposited deep in the lungs, causing interstitial fibrosis. Over 250,000 global deaths in 1990 and 2013 were attributable to pneumoconiosis. In spite of greatly improving preventative technologies and knowledge, deaths caused by pneumoconiosis from external airborne agents have not changed in over two decades [7].

Moreover, evidence exists for another potentially important route of exposure to external airborne agents, suggesting a possible mechanism through the olfactory nerve. In this mechanism, the external airborne agents precipitated in the nasal olfactory mucosa and directly translocated to the brain via the olfactory epithelium, which forms a direct connection between the nose and the brain along the axons of the olfactory nerve [8]. Distinct from quantitative studies that are focused on dysfunctions of the internal human organs, recent researchers have investigated the relationships between exposure to external airborne agents and various systemic ailments.

An area that has been of interest in the literature is the effect of the exposure to these agents on cognitive function and neurodegenerative diseases, such as Alzheimer’s disease and dementia [9,10]. Previous studies have examined the relationship between exposure to external airborne agents and decreased cognitive function [11,12,13,14,15], but the results have been inconsistent. Other investigations observed a significant association of exposure to external airborne agents with dementia and Alzheimer’s disease [16]. A review suggested a plausible mechanism demonstrating the effect of external airborne agents on cognitive dysfunction that can explain the loss of neural function [9]. Toxicological studies have postulated empirical evidence revealing the relationship between external airborne agents and neurodegeneration and proposing potential biological pathways [17].

However, there is limited information about the risk of neurodegenerative diseases with respect to exposure to external airborne agents [18]. Therefore, this study aimed to estimate the effect of external airborne agents’ exposure on dementia in a targeted Korean general population.

## 2. Methods

### 2.1. Data and Study Participants

This analysis used data from the National Health Insurance Service-Health Screening Cohort (NHIS-HealS, 2002–2015) collected by the National Health Insurance Service (NHIS) in the Republic of Korea [19]. The Korean NHIS supplies mandatory public health insurance to approximately 98% of the citizens residing within the territory of Korea. These national representative medical insurance-based data were provided to public health professionals and policy makers after the object-focused modification of five categories: the NHIS-national sample cohort; the NHIS-Senior; the NHIS-Female employees; the NHIS-Infants and children’s health screening; and the NHIS-HealS [20]. The NHIS-HealS represented national representative medical check-up participants, comprising a dataset that included socioeconomic demographics, type of insurance, medical facility visit information, and health examination results from 2002 to 2015. It was based on randomly selected participants comprising 10% of approximately 5.1 million health screening participants between 2002 and 2003 [21].

**Figure 1:**
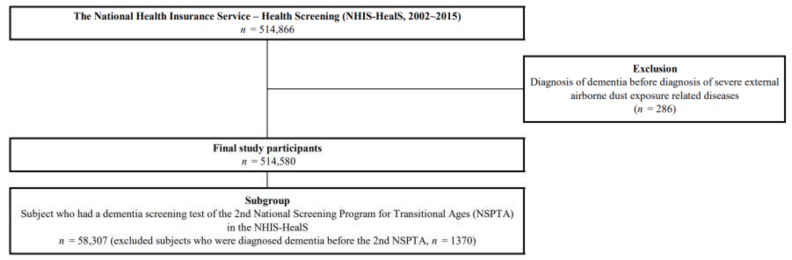
Schematic diagram depicting study population.

We selected 514,580 participants from the NHIS-HealS, excluding 286 individuals who were diagnosed with dementia before being diagnosed with severe external airborne agents’ exposure-related diseases. Furthermore, we used the subgroup data from the second National Screening Program for Transitional Ages (NSPTA) of the NHIS-HealS. The NSPTA is conducted to evaluate the physical and the mental health status of 40- and 66-year-old populations, who are in the key transition periods of their lifecycle. The second NSPTA was conducted for 66-year-olds and used results of the Korean Dementia Screening Questionnaire-Cognition (KDSQ-C). We selected participants who completed the second NSPTA in the NHIS-HealS (*n* = 58,307), after excluding those diagnosed with dementia before the second NSPTA (*n* = 1370). The detailed study flow demonstrated in Figure 1.

### 2.2. Dementia

Dementia was defined based on the medical records obtained from the NHIS-HealS. The NHIS-HealS had medical facility visit information with diagnosis codes based on the International Classification of Diseases 10th revision (ICD-10) from the standardized protocol of the Korean Classification of Diseases and Causes of Death 4th edition [22]. The dementia group included participants who had visited a medical facility with any of the following ICD-10 codes in the follow-up periods: “F00 Dementia in Alzheimer’s disease,” “F01 Vascular dementia,” “F02 Dementia in other diseases classified elsewhere,” or “F03 Unspecified dementia.”

Dementia screening test was evaluated using the KDSQ-C short form that includes five questions assessing memory impairment [23]. Response options of “never”, “sometimes”, and “frequently” are scored as 0, 1, and 2, respectively, and the cut-off values for dementia was a sum score >4 [24]. A score >4 in the KDSQ-C was defined as positive in the second NSPTA during the follow-up period.

### 2.3. Exposure to Severe External Airborne Agents

The NHIS-HealS or other modified dataset from the NHIS did not contain completed occupational and environmental information, including external airborne agents. Therefore, we defined the external airborne agents’ exposure based on the most severe cases of this type of exposure. The ICD-10 codes J60–70 comprise 11 subgroups: J60, coal-worker pneumoconiosis; J61, pneumoconiosis due to asbestos and other mineral fibers; J62, pneumoconiosis due to dust containing silica; J63, pneumoconiosis due to other inorganic dusts; J64, unspecified pneumoconiosis; J65, pneumoconiosis associated with tuberculosis; J66, airway disease due to specific organic dust; J67, hypersensitivity pneumonitis due to organic dust; J68, respiratory conditions due to inhalation of chemicals, gases, fumes, and vapors; J69, pneumonitis due to solids and liquids; and J70, respiratory conditions due to other external agents. They were categorized as “lung diseases due to external agents” by the World Health Organization (WHO) [25]. We excluded “J69, pneumonitis due to solids and liquids” from the severe external airborne agents’ exposure definition because solids and liquids were considered different from external airborne agents. We defined severe external airborne agents’ exposure group as patients with ICD-10 codes from J60 to J70, except J69, from the NHIS-HealS in the follow-up periods. It indicated a severe respiratory abnormal outcome closely related to exposure to external airborne agents as seen in previous study [26].

### 2.4. Other Covariates

Socioeconomic status included sex, age groups (~50, 51–60, and >60 years) and five household income groups (lowest, moderate-low, moderate, moderate-high, and highest) at baseline. The subgroup (second NSPTA data) characteristics included history of cerebral vascular disease, cardiovascular disease, smoking (never or past and current), and drinking (never or moderate and severe), and body mass index (BMI). BMI was categorized into three groups based on the Asian standard: underweight (<18.5 kg/m^2^), normal weight (<25 kg/m^2^), and overweight (≥25 kg/m^2^).

### 2.5. Statistical Analysis

A chi-squared test was conducted to describe differences in characteristics between the groups with and without dementia and the results of the dementia screening test. To estimate the effect of severe external airborne agents’ exposure on the incidence of dementia, we used the indirect age-standardized incidence ratio (SIR) and 95% confidence interval (CI) of dementia using a reference group of all participants of the NHIS-HealS. To demonstrate the risk of preclinical dementia related to severe external airborne agents’ exposure, the logistic regression analysis was used to estimate the risk of testing positive in the dementia screening test, adjusted for age at baseline, sex, household income, history, and health behavioral status according to the severe external airborne agents’ exposure with participants of the second NSPTA during the follow-up period. All analyses were conducted using SAS (Statistical Analysis System), version 9.4 (SAS Institute, Cary, NC, USA).

### 2.6. Ethical Consideration and Consents for Publication

All data from the NHIS-HealS were collected with written informed consent from all participants by the NHIS of the Republic of Korea, and the data were anonymized. The Institute Review Board (IRB) of the Gil Medical Center, Gachon University approved this study (IRB number: GCIRB2020-070).

## 3. Results

The characteristics of participants of the NHIS-HealS are summarized in Table 1. The total number of participants was 514,580, and 26,050 participants (5.1%) had been diagnosed with dementia. There were significant differences between participants with dementia and those without dementia with respect to sex, age, household income level, and severe external airborne agents’ exposure (*p* < 0.0001). Of the total number of participants, 1340 (0.3%) experienced severe external airborne agents’ exposure, and 111 participants (8.3%) had been diagnosed with dementia.

Table 2 shows the age-SIR and 95% CI of dementia in participants with severe external airborne agents’ exposure. Severe external airborne agents’ exposure was significantly associated with different types of dementia. The SIRs of dementia in Alzheimer’s disease, vascular dementia, dementia in other diseases, and unspecified dementia were 1.24 (1.01–1.49), 0.88 (0.37–1.32), 1.16 (0.01–2.77), and 0.69 (0.36–1.02), respectively.

To further explore the association between severe external airborne agents’ exposure and dementia, we analyzed participants of the second NSPTA according to the results of the dementia screening test. The characteristics of the participants are summarized in Table 3. The total number of the participants was 58,307, and 9844 participants (16.9%) tested positive in the dementia screening test. The participants were categorized based on sex, age groups, household income level, cerebral vascular disease, cardiovascular disease, BMI, smoking habit, drinking habit, and exposure to severe external airborne agents. All factors showed a significant difference between the participants testing positive and negative in the screening test, except for household income level, BMI, and drinking.

Table 4 presents the logistic regression analysis results of the positive dementia screening test among participants with severe external airborne agents’ exposure according to confounding factors (Model 1: age, sex, and household income level; Model 2: all statuses from Table 3). All models revealed that the risk of dementia significantly increased with external airborne agents’ exposure. The odds ratios with 95% CI of Crude model and Models 1 and 2 were 1.45 (1.04–2.03) and 1.42 (1.02–1.98), respectively.

Further results of the effect of severe external airborne dust exposure on the incidence of dementia and of the risk of positive screening test of dementia in supplementary tables.

## 4. Discussion

This study demonstrated that severe external airborne agents’ exposure was significantly associated with a high risk of dementia, and a high association was noted for dementia in Alzheimer’s disease. In a logistic regression analysis based on a dementia screening test, severe external airborne agents’ exposure revealed a significantly increased risk.

The observed association between exposure to airborne agents and dementia was consistent with the results of a previous study. Jung et al. reported a 138% increased risk of Alzheimer’s disease due to particle matter exposure [10]. Another study assessed the potential impact of particle matter exposure on dementia and neurodegenerative diseases such as Alzheimer’s disease. They reported a significant association between both diseases and exposure to particle matter: hazard ratios of 1.08 for dementia and 1.15 for Alzheimer’s disease [16]. However, these two studies were conducted on participants >65 years and focused on the effect of air pollution.

**Figure 2:**
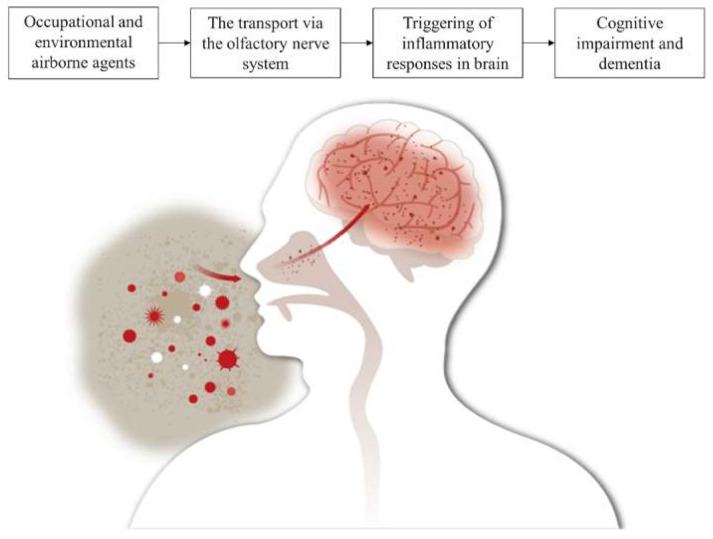
The mechanism of cognitive impairment and dementia from inhaled external airborne agents.

Some brain-related scientific studies could explain the possible mechanisms of how inhalation of external airborne agents may affect the cognitive function. Small particles from the external agent may repeatedly stimulate the olfactory system and cause systemic inflammation. Recent studies have reported that chronic systemic inflammation is associated with a decrease in cognition-related functions, such as processing speed [27] and memory [28]. The chronic systemic inflammation activates microglia and astrocytes in the brain, producing pro-inflammation and gradually increasing the level of neuroinflammation [29]. The high level of neuroinflammation has been known to cause hippocampal atrophy [30,31] and abnormal substantia nigra activity [32]. The mechanism of cognitive impairment and dementia from inhaled external airborne agents is demonstrated in Figure 2.

The severe external airborne agents’ exposure group comprised individuals diagnosed with airborne agents-related disease acquired from the environment. Most of the severe external airborne agents included respiratory hazardous factors at the workplace such as asbestos, mineral fibers, dust containing silica, chemicals, gases, fumes, and vapors. Airborne agents usually generate complex mixtures during occupational activities. These airborne agents from the workplace have a synergistic effect on burden diseases [33]. The WHO reported that a large number of workers are exposed to occupational dust at their workplace globally [34]. Due to the improved protective technology, the working environment could be ameliorated. However, dusts are ubiquitous in occupational activities and ordinarily neglected, thereby making it difficult for workers to escape the exposure to occupational dusts, who then remain unaware of their exposure in the workplace.

This study had a few limitations. Although it demonstrated a high association between severe external airborne agents’ exposure and dementia using a survey-based screening test with a large population, further medical information such as neuroimaging research might be necessary to strengthen the evidence of these results. This study only demonstrated the association between the risk of dementia and the airborne agents’ exposure group, which included only those patients with extremely severe airborne agents’ exposure. Thus, these findings may be somewhat limited to be generalized to the average general population. Another source of uncertainty is the lack of information about the exposure level of airborne agents. Detailed concentration of airborne agents is needed to understand the dose-response relationship between dust exposure and dementia. Further studies with more accurate and detailed information about workplace environment, concentration of occupational dusts, and co-exposure are required. We conducted a subgroup analysis of the effect of external airborne agents’ exposure on dementia according to type of severe external airborne agents’ exposure in supplementary Tables S1 and S2. However, further analyses, such as the interaction effect or a tree-based analysis between severe external airborne agents’ exposure and other covariates, were not conducted. A further study with more focus on effect of exposure to severe external airborne agents is, therefore, suggested.

## 5. Conclusions

In conclusion, this study found that severe external airborne agents’ exposure is a potential risk factor for dementia, especially in Alzheimer’s disease. This relationship was further elicited by the results of the dementia screening test. Further epidemiological, cohort, and clinical evidences are required. It is essential to create international awareness by continuing dementia research related to airborne agents exposure as well as continuing developing management strategies to protect individuals against severe external airborne agents’ exposure.

## Figures and Tables

**Table 1:** Baseline characteristics of the National Health Insurance Service-Health Screening (2002–2015) participants according to dementia.

	Total Participants, *n* (% of Column)	Dementia, *n* (% of Row)		*p*-Value
		No	Yes	
Total participants	514,580 (100.0)	488,530 (94.9)	26,050 (5.1)	
Sex				<0.0001
Male	278,967 (54.2)	269,131 (96.5)	9836 (3.5)	
Female	235,613 (45.8)	219,399 (93.1)	16,214 (6.9)	
Age (years) at baseline				<0.0001
~50	236,960 (46.0)	235,987 (99.6)	973 (0.4)	
51–60	144,987 (28.2)	140,954 (97.2)	4033 (2.8)	
>60	132,633 (25.8)	111,589 (84.1)	21,044 (15.9)	
Severe external airborne dust exposure				<0.0001
No	513,240 (99.7)	487,301 (95.0)	25,939 (5.0)	
Yes	1340 (0.3)	1229 (91.7)	111 (8.3)	

**Table 2:** Age-standardized incidence ratio and 95% confidence intervals of dementia by severe external airborne dust exposure.

	Incidence (Cases, %)		SIR (95% CI)
	Severe External Airborne Dust Exposure		
	No	Yes	
Dementia (F00-03) *	25,939 (5.0)	111 (8.3)	1.04 (0.85–1.24)
Dementia in Alzheimer’s disease (F00)	15,828 (3.0)	80 (6.0)	1.24 (1.01–1.49)
Vascular dementia (F01)	3541 (0.7)	12 (0.9)	0.88 (0.37–1.32)
Dementia in other diseases classified elsewhere (F02)	428 (0.1)	2 (0.1)	1.16 (0.01–2.77)
Unspecified dementia (F03)	6142 (1.2)	17 (1.3)	0.69 (0.36–1.02)

SIR: age-standardized incidence ratio. CI: confidenc interval. * These codes are the ICD-10 codes.

**Table 3:** Baseline characteristics of second National Screening Program for Transition Ages participants by dementia screening results.

	Total Participants *n* (% of Column)	Dementia Screening Test *n* (% of Row)		*p*-Value
		Negative	Positive	
Total participants	58,307 (100.0)	48,463 (83.1)	9844 (16.9)	
Sex				<0.0001
Male	29,018 (49.7)	24,870 (88.7)	4148 (14.3)	
Female	29,289 (50.3)	23,593 (80.6)	5696 (19.5)	
Age (years) at baseline				<0.0001
~60	43,698 (74.9)	37,313 (85.4)	6385 (14.6)	
>60	14,609 (25.1)	11,150 (76.3)	3459 (23.7)	
Household income level				0.4097
Lowest	10,685 (18.3)	8917 (83.5)	1768 (16.5)	
Moderate-low	10,679 (18.3)	8847 (82.9)	1832 (17.1)	
Moderate-high	18,208 (31.2)	15,003 (82.4)	3205 (17.6)	
Highest	18,735 (32.2)	15,696 (83.8)	3039 (16.2)	
Cerebral vascular disease				<0.0001
No	55,648 (95.4)	46,527 (83.6)	9121 (16.4)	
Yes	2659 (4.6)	1936 (72.8)	723 (27.2)	
Cardiovascular disease				<0.0001
No	53,519 (91.8)	44,714 (83.6)	8805 (16.5)	
Yes	4788 (8.2)	3749 (78.3)	1039 (21.7)	
Body mass index				0.0545
Underweight (<18.5 kg/m2)	1122 (1.9)	917 (81.7)	205 (18.3)	
Normal (18.5–24.9 kg/m2)	35,112 (60.2)	29,125 (82.9)	5987 (17.1)	
Overweight (≥25 kg/m2)	22,073 (37.9)	18,421 (83.5)	3652 (16.5)	
Smoking				0.0003
Never or past	51,729 (88.7)	42,892 (82.9)	8837 (17.1)	
Current	6578 (11.3)	5571 (84.7)	1007 (15.3)	
Drinking				0.3095
Never or moderate	51,121 (87.7)	42,460 (83.1)	8661 (16.9)	
Severe	7186 (12.3)	6003 (83.6)	1183 (16.5)	
Severe external airborne dust exposure				0.0011
No	58,084 (95.6)	48,304 (82.8)	9800 (17.2)	
Yes	223 (0.4)	167 (74.9)	56 (25.1)	

**Table 4:** Logistic regression results of positive dementia screening test according to occupational dust exposure group.

Severe External Airborne Dust Exposure	Odds Ratio (95% Confidence Interval)	
	Model 1	Model 2
No	Reference	Reference
Yes	1.45 (1.04–2.03)	1.42 (1.02–1.98)

Model 1 adjusted for age, sex, and household income level; Model 2 adjusted of age, sex, household income level, cerebral vascular disease, cardiovascular disease, body mass index, smoking, and drinking status.

## Supplementary Materials

The following are available online at https://www.mdpi.com/2077-0383/9/12/4069/s1, Table S1: Age-standardized incidence ratio and 95% confidence intervals of dementia according to type of severe ex-ternal airborne dust exposure, Table S2: Logistic regression results of positive dementia screening test according to type of severe external airborne dust exposure.
