# Supplementary material for: Effect of Severe External Airborne Agents’ Exposure on Dementia
Source: J Clin Med. 2020 Dec 17;9(12):4069. doi: 10.3390/jcm9124069 (PMC7766205; doi:10.3390/jcm9124069)
Supplement: Supplementary file 1 [file jcm-09-04069-s001.pdf]

Table S1. Age-standardized incidence ratio and 95% confidence intervals of dementia according to type of severe external airborne dust exposure.

	Dementia		SIR (95% CI)
	No	Yes	
Severe external airborne dust exposure (J60-70, except J69)*	1,229 (91.7)	111 (8.3)	1.04 (0.85–1.24)
J60 Coalworker pneumoconiosis	369 (90.9)	37 (9.1)	0.93 (0.63–1.24)
J61 Pneumoconiosis due to asbestos and other mineral fibbers	107 (89.2)	13 (10.8)	1.18 (0.54–1.82)
J62 Pneumoconiosis due to dust containing silica	49 (100.0)	0 (0.0)	-
J63 Pneumoconiosis due to other inorganic dusts	11 (84.6)	2 (15.4)	1.86 (0.01–4.44)
J64 Unspecified pneumoconiosis	300 (91.7)	27 (8.3)	0.91 (0.57–1.25)
J65 Pneumoconiosis associated with tuberculosis	22 (84.6)	4 (15.4)	1.26 (0.03–2.50)
J66 Airway disease due to specific organic dust	10 (90.9)	1 (9.1)	1.16 (0.01–3.42)
J67 Hypersensitivity pneumonitis due to organic dust	127 (92.0)	11 (8.0)	1.52 (0.62–2.42)
J68 Respiratory conditions due to inhalation of chemicals, gases, fumes and vapors	126 (91.3)	12 (8.7)	1.87 (0.81–2.94)
J70 Respiratory conditions due to other external agents	108 (96.4)	4 (3.6)	0.53 (0.02–1.05)

SIR: Age-standardized incidence ratio; CI: Confidence interval; *These codes are the ICD-10 codes.

Table S2: Logistic regression results of positive dementia screening test according to type of severe external airborne dust exposure.

	Dementia screening test		OR (95% CI)
	Negative	Positive	
Severe external airborne dust exposure (J60-70, except J69)*	167 (74.9)	56 (25.1)	1.42 (1.02–1.98)
J60 Coalworker pneumoconiosis	55 (67.1)	27 (32.9)	1.97 (1.19–3.26)
J61 Pneumoconiosis due to asbestos and other mineral fibbers	13 (76.5)	4 (23.5)	1.21 (0.33–4.41)
J62 Pneumoconiosis due to dust containing silica	8 (100.0)	0 (0.0)	-
J63 Pneumoconiosis due to other inorganic dusts	2 (100.0)	0 (0.0)	-
J64 Unspecified pneumoconiosis	37 (80.4)	9 (19.6)	1.03 (0.48–2.25)
J65 Pneumoconiosis associated with tuberculosis	3 (100.0)	0 (0.0)	-
J66 Airway disease due to specific organic dust	2 (100.0)	0 (0.0)	-
J67 Hypersensitivity pneumonitis due to organic dust	17 (81.0)	4 (19.0)	0.66 (0.15–2.90)
J68 Respiratory conditions due to inhalation of chemicals, gases, fumes and vapors	10 (58.8)	7 (41.2)	2.56 (0.87–7.55)
J70 Respiratory conditions due to other external agents	20 (80.0)	5 (20.0)	0.89 (0.31–2.64)

Results were after adjusting of age, sex, household income level, cerebral vascular disease, cardiovascular disease, body mass index, smoking, and drinking status; OR: Odds Ratio, CI: Confidence interval, *These codes are the ICD-10 codes.
